# Home Health Care Clinicians’ Use of Judgment Language for Black and Hispanic Patients: Natural Language Processing Study

**DOI:** 10.2196/42552

**Published:** 2023-04-17

**Authors:** Maxim Topaz, Jiyoun Song, Anahita Davoudi, Margaret McDonald, Jacquelyn Taylor, Scott Sittig, Kathryn Bowles

**Affiliations:** 1 Columbia University School of Nursing New York, NY United States; 2 Data Science Institute, Columbia University New York, NY United States; 3 Center for Home Care Policy & Research VNS Health New York, NY United States; 4 Department of Health Sciences University of Louisiana at Lafayette Lafayette, LA United States; 5 Department of Biobehavioral Health Sciences University of Pennsylvania School of Nursing Philadelphia, PA United States

**Keywords:** stigmatizing language, judgment language, health disparity, natural language processing, home health care, nursing informatics, racial biases, language barrier, cohort study, racial difference

## Abstract

**Background:**

A clinician’s biased behavior toward patients can affect the quality of care. Recent literature reviews report on widespread implicit biases among clinicians. Although emerging studies in hospital settings show racial biases in the language used in clinical documentation within electronic health records, no studies have yet investigated the extent of judgment language in home health care.

**Objective:**

We aimed to examine racial differences in judgment language use and the relationship between judgment language use and the amount of time clinicians spent on home visits as a reflection of care quality in home health care.

**Methods:**

This study is a retrospective observational cohort study. Study data were extracted from a large urban home health care organization in the Northeastern United States. Study data set included patients (N=45,384) who received home health care services between January 1 and December 31, 2019. The study applied a natural language processing algorithm to automatically detect the language of judgment in clinical notes.

**Results:**

The use of judgment language was observed in 38% (n=17,141) of the patients. The highest use of judgment language was found in Hispanic (7,167/66,282, 10.8% of all clinical notes), followed by Black (7,010/65,628, 10.7%), White (10,206/107,626, 9.5%), and Asian (1,756/22,548, 7.8%) patients. Black and Hispanic patients were 14% more likely to have notes with judgment language than White patients. The length of a home health care visit was reduced by 21 minutes when judgment language was used.

**Conclusions:**

Racial differences were identified in judgment language use. When judgment language is used, clinicians spend less time at patients’ homes. Because the language clinicians use in documentation is associated with the time spent providing care, further research is needed to study the impact of using judgment language on quality of home health care. Policy, education, and clinical practice improvements are needed to address the biases behind judgment language.

## Introduction

Home health care is one of the fastest-growing outpatient settings in the United States, where about 200,000 clinicians (including registered nurses, physical or occupational therapists, and social workers) treat more than 5 million patients annually [1,2]. During home health care, clinicians treat patients’ conditions (eg, wounds), provide health promotion interventions (eg, self-management education), and assist with medication management and reconciliation [3]. About 25% of home health care patients represent a racial and ethnic minority population (eg, Latinx and Black patients), which is higher than the number of minority patient populations in other outpatient settings [1].

Although quality of care is affected by numerous factors (eg, structural resources, levels of clinician education, and patient-per-clinician-ratios) [4-6], a clinician’s biased behavior toward patients (such as evaluating one group and its members negatively relative to another) can affect the quality of care [7]. Recent literature reviews [8-10] report on widespread implicit biases among clinicians. For example, a recent review of 215 studies [11] showed that, most commonly, nurses exhibit biases in the area of race and ethnicity. These biases affect clinicians’ behaviors and care decisions regarding their patients, affecting patient adherence and outcomes [12-14].

In home health care, health disparities are well documented, with Black and Hispanic patients receiving a lower quality of care [15-18] and having worse outcomes (eg, higher hospitalization rates) [19-23] compared to White patients. In light of growing recognition of the effects of racism on health disparities and inequities, reducing racial biases has become a key priority for many health care organizations around the United States [24-26].

Emerging studies in hospital settings show racial biases in the language used in clinical documentation within electronic health records. Specifically, several recent studies [27-29] used natural language processing (a computer science–based method that can help extract meaning from a large corpus of text) to search for instances of stigmatizing language and then compared the prevalence of stigmatizing language by race and ethnicity. A specific example of stigmatizing language is judgment language, conveying disbelief in patients’ statements [27-29]. Other examples include using negative descriptors when referring to marginalized patients [27-29]. These recent studies found that clinical notes written about Black patients had 25%-50% higher odds of containing stigmatizing language than the notes written about White patients [27-29]. Some studies have also started to explore associations between stigmatizing language and quality of care. For example, a recent study showed that exposure to stigmatizing language in clinical notes is associated with more negative attitudes toward the patient and less aggressive management of the patient’s pain [30].

Of note, stigmatizing language was reported to also be more frequent in the documentation of patients with substance use disorder and certain chronic conditions (eg, diabetes) [29]. Other potential factors that can affect clinical documentation quality and the use of specific language include the patient’s culture (eg, Asian) [31], age [32], and clinical complexity [29]. This study focuses on associations between stigmatizing language and race and ethnicity in clinical documentation.

Our extensive literature search identified no studies investigating the extent of stigmatizing language in home health care. To bridge the gaps in the literature, this study aimed to understand how stigmatizing language might manifest in home health care electronic health records and whether the presence of stigmatizing language might be associated with quality of care. First, we developed a natural language processing system to detect the presence of a specific type of stigmatizing language—judgment language—in home health care clinical notes and explore racial differences in using such language in a racially diverse patient sample. Second, we explore the association between the use of judgment language and time spent by home health care clinicians in a patient’s home as a reflection of care quality.

## Methods

### Study Setting

We used data extracted from a large urban home health care organization in Northeastern United States. The home health care agency provides skilled home health care services, including nursing, physical and occupational therapy, and social work.

### Ethics Approval

The study was approved by the institutional review board of the participating organization, VNS Health (IRB I22-001).

### Study Data Set

This study examined data collected during routine home health care services between January 1 and December 31, 2019. All data were extracted from the home health care agency’s electronic health record system. The data included the patient’s sociodemographic information, specifically the patient’s gender and race or ethnicity, collected using a federally mandated assessment data set called the “Outcome and Assessment Information Set” (OASIS) that captures race or ethnicity with the following categories: Asian, White, Black, Hispanic, and other (eg, Native Hawaiian or Pacific Islander). Since only a small number of patients identified as “other” race or ethnicity, we removed this group from this analysis. Although the OASIS allows for multiselect in race and ethnicity, to establish mutually exclusive groups, if Hispanic was one of the selections, then the individual was categorized in the Hispanic group. In addition, we extracted narrative clinical notes, clinician information (ie, clinician author ID for each narrative note), and the length of time spent by a clinician in the patient’s home (in minutes). Home health care clinicians in this study included nurses, physical or occupational therapists, and social workers.

Narrative notes were visit notes that home health care clinicians used to document the patient’s symptoms and health care inventions that occurred during home visits. Overall, we extracted 264,146 visit notes documented for 45,384 patients, with an average of 6 visit notes per patient. Visit note average length was 298 characters, corresponding to about 4-6 sentences.

### Language Suggesting Judgment of Patients

Based on previous literature [27,28], we identified a specific type of language that potentially suggests judgment of patients—“judgment words.” Judgment words allow the speaker to distance themselves from the source of knowledge and directly question the speaker’s credibility. The initial list of judgment words was extracted from previous literature and included the following terms: “adamant,” “apparently,” “claims,” “insists,” and “states” [27].

In previous work [27], the use of quotes was also found to indicate judgment of patient’s claims; for example, “the patient reports she had a ‘reaction’ to the medication.” However, in home health care clinical notes, we found that clinicians use quotes very infrequently. Hence, we decided to omit this category from the analysis.

### Natural Language Processing System Development

**Figure 1:**
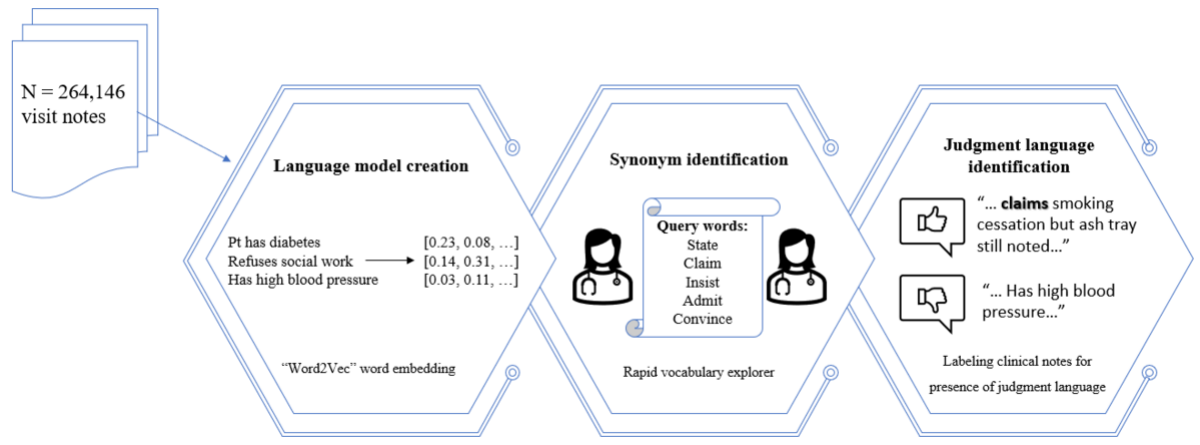
A diagram of the development of a natural language processing system. Pt: patient.

We used our previously developed and validated open-source natural language processing system, NimbleMiner [33,34], to expand the initial vocabularies of evidential and judgment words. Specifically, NimbleMiner was implemented in 3 steps that are briefly described below and captured in Figure 1 (a complete software architecture description is available elsewhere [33,34]).

#### Step 1: Language Model Creation

Language models are numerical representations of semantic and lexical associations between words and expressions in large bodies of text. We generated a language model (word embedding model called word2vec) [35] using all home health care clinical notes available in our sample (N=264,146 visit notes).

#### Step 2: Synonym Identification

Using NimbleMiner’s “Rapid vocabulary explorer” module, we queried the language model for synonyms and other lexical variants (eg, misspellings) of terms that indicate judgment language. For example, querying the language model for synonyms of the word “claims” results in the system presenting to the user a list of potentially similar terms, including synonyms such as “admits” and misspellings such as “claimes.” Two home health care experts conducted language model queries independently, and their synonym lists were merged and finalized by the study team via discussion.

#### Step 3: Automated Identification of Judgment Language in Clinical Notes

We searched all clinical notes for terms identified in step 2. At this step, we also modified the software to exclude irrelevant and negated terms from the set of clinical notes with positive matches. For example, we excluded clinical notes that included irrelevant expressions such as “vn [visiting nurse] convinced pt [patient],” “cardiologist stated,” and “primary care insisted.” Such language was excluded since the focus of this study was on expressions that clinicians use to describe the patient rather than other individuals, such as other clinicians. The final product of this step included all clinical notes labeled as either having or not having the language of judgment.

### Statistical Analyses

First, we used chi-square tests to conduct bivariate analyses to examine differences in the use of judgment language by patients’ race or ethnic groups. Further, we conducted an adjusted analysis to explore associations between the patients’ race or ethnic group and the use of judgment language in their clinical notes. To examine whether the analysis should be adjusted for individual clinicians’ writing style, we implemented and compared the results of two logistic regression models: (1) a general logistic regression model adjusted for patients’ gender and (2) a mixed-effects logistic regression model adjusted for patients’ gender (fixed effects) and clinician ID (random effects). The most appropriate model was selected by comparing differences in the log-likelihood between the models [36]. Finally, we examined the association between visit time and the use of judgment words. Specifically, we implemented linear regression to analyze this association adjusted for the patient’s gender and race or ethnic group. All analyses were implemented in Stata statistical software (version 17; StataCorp) [37].

## Results

Using the “Rapid vocabulary explorer” module of the natural language processing software (NimbleMiner), judgment language vocabulary was expanded to include additional terms (eg, “convinced,” “vehemently,” “believes,” and “admits”), linguistic variations of the judgment word terms (eg, “claim,” “claims,” and “claimed”), as well as misspellings (eg, “claimes,” “clamed,” and “clai med”). Table 1 provides examples of clinical notes that had judgment language.

**Table 1:** Examples of clinical notes with judgment language. Judgment words within the quotes are italicized.

Examples from clinical notes	Judgment language
“…*claims* smoking cessation but ash tray still noted on night stand.”	claims
“pt [patient] *claims* he had fever in past, but no thermometer in use.”	claims
“He has a rw [rolling walker] but pt [patient] only uses it to get up fr [from] the bed. pt demoed another safe method of getting out of the bed, but pt *insisted* of doing it on his own manner.”	insisted
“pt [patient] also *insisted* vn [visiting nurse] to remove left foot dressing however no wound order suggested to do so.”	insists
“has a rollator but husband is so *adamant* for pt [patient] not to use it.”	adamant
“Patient has a straight cane but *adamantly* refused it in the apt [apartment] and patient prefer holding on walls and furnitures.”	adamantly
“Patient *states* that she feels weak and dizzy patient *admits* to not testing blood sugars as ordered but *states* she takes her insulin.”	states, admits
“patient refuses to wash legs and *claims* he is allergic to water. patient *convinced* genetic medicine is only solution for his wound care treatment.”	convinced, claims
“pt [patient] has D.M. [diabetes mellitus] and H.F. [hear failure], but *convinced* they don’t need to keep low sugar diet.”	convinced
“s/p [status post] hospital d/c [discharge] where she was tx [treated] with hemodialysis after skipping 3 txs, as per d/c summary. pt *vehemently* denies this.”	vehemently
“Patient *admits* to not testing blood sugars as ordered but *states* she takes her insulin.”	admits, states

In total, judgment language was used for 17,141 patients, which is 38% of the overall patient sample. Further, 10% (26,306/264,146) of all clinical notes included judgment language. As presented in Table 2, there were significant differences in the distribution of judgment language in clinical notes by race or ethnicity. The lowest amount of judgment language was identified among Asian patients (1756/22,548, 7.8% of all notes had judgment language), followed by White (10,206/107,626, 9.5%), Black (7010/65,628, 10.7%), and Hispanic patients (7167/66,282, 10.8%). The relative increase in the proportion of notes with judgment language among Black and Hispanic patients reached about 14%, compared to White patients (*P*<.001). For Asian patients, the lowest rates of judgment language were observed.

**Table 2:** Distribution of judgment language by race or ethnicity.

Race or ethnicity	Total patients (N=45,384), n (%)	Total clinical notes (N=264,146), n	Clinical notes with judgment language (n=26,306), n (%)^a^	Relative change compared to White (%)	Odds ratios (95% CIs)
White	19,826 (44)	107,626	10,206 (9.5)	Reference	Reference
Asian	3921 (9)	22,548	1756 (7.8)	–18	0.91 (0.85-0.96)
Hispanic	10,503 (23)	66,282	7167 (10.8)	+14	1.05 (1.01-1.1)
Black	10,969 (24)	65,628	7010 (10.7)	+13	1.09 (1.04-1.14)

^a^*P*<.001.

In the adjusted analysis using logistic regression, the difference between racial or ethnic groups remained significant (*P*<.001). Specifically, Black and Hispanic patients had 5% and 9% (respectively) higher odds of judgment language presence than White patients (Table 2).

The random effect for clinician ID was significant, as indicated by comparing the log-likelihoods of regression models with and without the random effect for clinician ID. We found that removing the random effect causes a substantial drop in the log-likelihood (~20%), and the effect is statistically significant (*P*<.001). These results indicate that clinicians’ writing style was associated with judgment language. In other words, some clinicians use more judgment language than others.

On average, clinicians spent 1 hour 8 and minutes in patients’ homes. Further, clinicians spent 24 fewer minutes in the patient’s home when they used judgment language in clinical notes (46 minutes average home health care visit length) compared to when no judgment language was used (70 minutes average home health care visit length). In the further linear regression analysis adjusted for the patient’s race or ethnicity and gender, each judgment word was associated with a 21-minute decrease in the home health care visit time (CIs 22.9-19.9; *P*<.001).

## Discussion

### Principal Findings

This study was the first to investigate the use of stigmatizing language in home health care. Specifically, we developed and applied a natural language processing system that identified the language of judgment in clinical notes. We found that such language appeared relatively frequently in clinical notes, with nearly 40% of patients having at least one instance of such language in their notes. Overall, approximately 1 in 10 clinical notes included judgment language, which is similar to the previous literature in hospital settings [27,29,38]. These numbers highlight the need for further studies with larger data sets of clinical data that will enable estimating the general prevalence and use of judgment language in health care.

Further, our findings helped identify racial and ethnic differences in the use of judgment language. Previous studies primarily focused on language differences between Black and White patients [27,29,38], while our sample also included a significant number of Hispanic and Asian patients. We found that judgment language was more frequently documented in clinical notes of Black and Hispanic patients. Specifically, in an adjusted analysis, Black and Hispanic patients had up to 9% higher odds of having judgment language in their clinical notes than White patients. These results are lower but in the same direction as previous findings from the hospital settings, indicating that Black patients have up to 25% higher odds of having judgment language in their clinical notes than White patients [27]. We further expand these results and show that similar to Black patients, Hispanic patients have high levels of judgment language in clinical notes.

Several potential explanations can help describe these differences. First, clinicians’ personal biases might manifest in written documents [27-29]; hence, we find the language of judgment to be more prevalent in clinical notes of minority patient populations. Our analysis also shows that some clinicians are more likely to use the language of judgment than others. This further supports the need for more research to test the hypothesis that personal differences among clinicians play a significant role in shaping their language. In addition, institutional biases toward certain racial and ethnic groups might shape clinicians’ views, resulting in documentation differences. For example, numerous previous studies found that racist institutional policies lead to worse quality of care and outcomes among Black patients [10].

In addition to the previously mentioned factors that may contribute to the use of judgment language in clinical notes, it is important to consider the role of patient-provider interactions in shaping the use of this type of language. The use of judgment language may not be solely the result of clinician biases but may also be influenced by the specific circumstances of the patient-provider interaction. For example, when a patient is not following instructions or refusing self-management, a clinician may be more likely to use judgment language in their documentation. Similarly, in complex clinical scenarios, a clinician may use more judgment language as they navigate a difficult case in which diagnosing a patient’s condition is complicated. Further, in some clinical situations, clinicians might use harsher, more critical, or more negative language. Further research is needed to understand specific contextual factors that may contribute to the use of judgment language in clinical notes.

Finally, our results hint at the potential association between the language of judgment and quality of care. Specifically, we found that clinicians spend less time with patients for whom they document the language of judgment. This is concerning since shorter home health care visits are associated with a higher risk for poor outcomes (eg, higher risk of hospitalizations) [39,40]. Another testable hypothesis might be that home health care clinicians spend less time with patients they perceive negatively, which is reflected by judgment language.

Our findings have several implications at the health care policy and management levels. First, health care organizations might need to develop guidelines to help shape more inclusive and neutral documentation patterns. For example, certain words and expressions of judgment might need to be limited or require thorough justification when used. Further, targeted clinician training in improving documentation styles might be needed for some clinicians who frequently use such language in their documentation. In addition, counseling or educational interventions to reduce implicit clinician biases might help decrease stigmatizing language in clinical practice. Finally, clinicians might need more training about engaging with patients who are not following instructions or refuse self-management to increase time spent with those patients in productive motivational conversations and similar interventions [41].

### Limitations

This work has several significant limitations. First, the judgment language in the notes might appear in descriptions of “nonjudgmental” clinical situations. For example, words like “state(s)” are often used to describe patient’s symptoms and other reports with little evidence of judgment. Additionally, the study’s approach to identifying judgment language is based on the frequency of certain words rather than considering the context in which they are used. This means that the study may not accurately capture the nuances of how judgment language is used in clinical notes, and therefore, may not fully capture the extent to which clinical notes are racially charged. Other natural language processing methods computationally tied to the clinical note context (eg, sequence of words, topic modeling, or Bidirectional Encoder Representations from Transformers [BERT]) [42] might help identify the judgment language more accurately.

Second, this analysis did not adjust for clinical factors that might interfere with judgment language prevalence (eg, the patient’s cognitive status or mental health conditions). Further work is needed to generate comparisons adjusted for such additional health conditions.

Home health care visit length might not necessarily reflect the quality of care provided. Further, clinical encounter time as well as documentation time and quality might be affected by multiple factors, such as administrative concerns or needing to visit another patient’s home as soon as possible. Associations between clinicians’ documentation and encounter times should be explored in future studies.

Further work is needed to understand whether differences in judgment language prevalence exist between different disciplines (ie, nursing, occupational or physical therapy, and social work). In addition, further exploration of the effect of culture on the language used during and length of clinical encounters is needed. For example, Asian patients might respond differently during clinician interaction [43], which might affect clinician documentation. Further, future studies should emphasize the frequency of the judgment words used rather than focusing solely on the specific vocabulary used. This will test whether a higher frequency of judgment words in the clinical notes may indicate a more intentional use of judgmental language, as opposed to a lower frequency, which may suggest a less intentional use. Further qualitative research with the clinicians who wrote the clinical notes is needed to gain a deeper understanding of the use of judgment language and the context in which it was used. Finally, the generalizability of this work is limited to one, albeit large, home health care agency.

### Conclusions and Implications

This study’s findings indicate that language of judgment appears more frequently in clinical notes of Black and Hispanic patients as compared to White and Asian patients. We also found that clinicians spend less time in patients’ homes when judgment language is used. Since the language clinicians use in documentation is associated with care quality, policy and clinical practice steps are needed to address biases associated with racial and ethnic differences in the prevalence of judgment language. Further research is needed to fully understand the prevalence and root causes of stigmatizing language and to test interventions to eliminate their use.

## Abbreviations

BERT: Bidirectional Encoder Representations from Transformers
OASIS: Outcome and Assessment Information Set
